# Thermo-Chemo-Mechanical Modeling of Residual Stress in Unidirectional Carbon Fiber-Reinforced Polymers during Manufacture

**DOI:** 10.3390/ma17123040

**Published:** 2024-06-20

**Authors:** Rui Bao, Junpeng Liu, Zhongmin Xiao, Sunil C. Joshi

**Affiliations:** 1 School of Mechanical and Aerospace Engineering, Nanyang Technological University, Singapore 639798, Singapore; baor1001@163.com (R.B.); mscjoshi@ntu.edu.sg (S.C.J.); 2 College of Safety and Ocean Engineering, China University of Petroleum, Beijing 102249, China; liujp@cup.edu.cn

**Keywords:** composite processing, epoxy prepreg, viscoelastic modulus, curing strain

## Abstract

The application of carbon fiber-reinforced composite materials in marine engineering is growing steadily. The mechanical properties of unbonded flexible risers using composite tensile armor wire are highly valued. However, the curing process generates a certain amount of internal residual stress. We present a detailed analysis of epoxy resin laminates to assess the impact of thermal, chemical, and mechanical effects on the curing stress and strain. An empirical model that correlates temperature and degree of cure was developed to precisely fit the elastic modulus data of the curing resin. The chemical kinetics of the epoxy resin system was characterized using differential scanning calorimetry (DSC), while the tensile relaxation modulus was determined through a dynamic mechanical analysis. The viscoelastic model was calibrated using the elastic modulus data of the cured resin combining temperature and degree of the curing (thermochemical kinetics) responses. Based on the principle of time–temperature superposition, the displacement factor and relaxation behavior of the material were also accurately captured by employing the same principle of time–temperature superposition. Utilizing the empirical model for degree of cure and modulus, we predicted micro-curing-induced strains in cured composite materials, which were then validated with experimental observations.

## 1. Introduction

**Figure 1:**
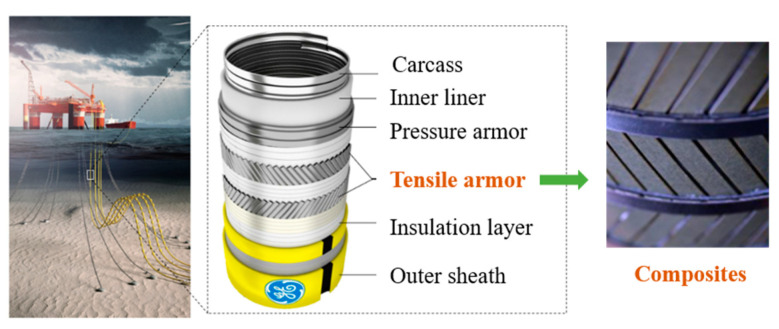
Structure of unbounded flexible risers in deep water.

As the exploration of offshore oil and gas resources extends into deeper waters, there is an increasing demand for high-performance equipment and longer transmission pipelines. This necessitates pipelines with high-pressure resistance (over 10,000 psi), a wide operating temperature range (−20 °C to +150 °C), excellent corrosion resistance, and high durability (with a service life exceeding 25 years). Furthermore, the pipelines must be capable of withstanding high flow rates, minimizing the environmental impact, and operating effectively at substantial depths (greater than 1000 m). Traditional unbonded flexible risers, typically reinforced with carbon steel tensile armor layers, are increasingly proving insufficient for deepwater operations due to their high density and significant weight. These armor layers, crucial for the structural integrity of the risers, consist of several flat steel bars spirally wound at angles between 30° and 50° relative to the axial direction. To maintain torsional and load balance, the adjacent armor layers are configured in reverse spirals with large angles. The main function of the tensile armor layers is to resist the axial tension and reduce the partial torque, ensuring that the riser remains operational and safe under its own weight and additional tensile force [1,2]. The riser structure is composed of multiple independent layers, each made from different materials and possessing unique sectional shapes, as shown in Figure 1.

The replacement of traditional steel with composite materials for manufacturing tensile armor layers presents a promising, yet under-researched, solution. Carbon fiber-reinforced polymers (CFRPs) are particularly suitable for this application based on their high strength, low weight, and corrosion resistance. CFRPs weigh approximately 77% less than traditional steel, which makes it an excellent choice for reducing the overall weight of flexible risers while maintaining the same load-bearing capacity. They possess an elevated strength-to-weight ratio, which enhances the structural efficiency of flexible risers by reducing the overall weight while maintaining the same load-bearing capacity. Additionally, CFRPs demonstrate superior fatigue and corrosion resistance. Extensive research on curing-induced thermal residual stress in composite materials has been conducted. However, few studies have specifically addressed the impact of residual stress on the strength of flexible riser composite tensile armor wires. A simple and effective computational model is needed to calculate the residual stress and strength failure in these composite materials. To do this, it is necessary to combine experimental research with numerical simulation through methods considering constitutive models, volume fraction, curing temperature, and service temperature of fibers and matrices. This will help establish a comprehensive computational theory and a calculation model for residual stress in composite tensile armor layers.

Compared to other materials, composite tensile armor wires present fewer inconveniences such as delamination, degumming, and voids, especially when manufactured using an autoclave. Pultrusion is a continuous manufacturing process for composite profiles, which significantly affects the quality and performance of the final product [3]. Due to the inherent anisotropy and heterogeneity of composite materials, combined with the mismatch in the thermal expansion coefficients between the internal fibers and the resin, residual stresses can develop. However, the residual stresses generated during the autoclave curing process can significantly impact the mechanical properties of the composites, potentially initiating matrix cracks, delamination, and warpage. These stresses may compromise the performance of the composite structure [4]. Dusi [5] analyzed the cure kinetics and viscosity of Fiberite 976 resin, determining the heat of reaction, rate of cure, and degree of cure at various temperatures using DSC and measuring viscosity over time with a viscometer. Mijović [6] analyzed the cure kinetics of three epoxy formulations composed of TGDDM and DDS using isothermal tests. They found that an auto-catalyzed mechanism with an overall reaction order of 2 adequately described the cure kinetics, noting increased reaction rates at higher temperatures and DDS concentrations. Fu [7], Che [8], and Singleton [9] analyzed cure deformations and residual stresses in 3D braided composites using a representative volume element (RVE) and thermochemical and thermodynamic models. They examined the effects of temperature rise rate and curing temperature on the degree of cure, deformation, and residual stresses. Therefore, it is crucial to consider these residual stresses in the design and engineering of composite material structures to ensure long-term reliability and performance.

Several factors affect the formation of residual stress and strain throughout the autoclave curing process. The accurate prediction of cure-induced residual stresses in composite laminates depends on a suitable constitutive model that incorporates factors such as thermal expansion, cure-induced chemical shrinkage, and material degradation or relaxation during curing. Traditionally, studies on residual stress have primarily been focused on elastic models during the cool-down stage only. These models overlook several key effects: chemical shrinkage, stress development before cooling, and stress relaxation during the cooling process. Studies have shown that the classic lamination theory (CLT) or its modified versions can effectively forecast the residual stress in thin laminates [10,11,12]. Stango and Wang [13] calculated thermal residual stresses by using CLT; they found that CLT overestimates residual stresses and discussed potential reasons for this discrepancy. However, these models do not account for residual stress development before cooling and fail to solve the complex coupling of temperature and curing degree in thick laminates. Consequently, an increasing number of scholars are paying attention to this critical issue.

It is important to recognize that most polymers, due to their viscoelastic properties like stress relaxation and strain creep, are not described by linear elastic models, particularly under conditions of high temperature and low degree of cure. Initially, Schapery [14] conducted series analyses on the viscoelastic stress of composite laminates. Following this, several researchers focused on studying residual stresses using viscoelastic models, predominantly during the cooling stage of the curing process [15]. Accurately simulating the viscoelastic behavior of materials throughout the entire curing process of laminated boards remains challenging. Subsequent studies incorporated factors such as the thermal expansion coefficient, cure-induced chemical shrinkage, and material stress relaxation, examining some fundamental characteristics of the matrix using the time–temperature superposition and equivalence principles [16,17,18,19]. Kim and White [20] investigated the stress relaxation behavior of 3501-6 epoxy resin during curing, using experimental data to model the relaxation process. Eom [21] applied time–temperature superposition to predict the instantaneous viscoelastic properties during cure, providing a method to anticipate material behavior throughout the curing process. Prasatya [22] developed a viscoelastic model to predict the isotropic residual stress in thermosetting materials, examining the effects of the processing parameters on stress development. As the thermal, physical, rheological, and mechanical properties of a resin change during curing, the analysis becomes more complex. To better represent these performance changes, advancements in modeling and further progress in experimental characterization are necessary.

In this paper, the reaction kinetics of CFRPs produced from unidirectional (UD) prepregs was characterized and modeled. The curing kinetics parameters of the material were obtained by non-isothermal differential scanning calorimetry (DSC). Additionally, a viscoelastic residual stress model for composite materials was developed, employing the time–temperature superposition principle to describe how the material’s mechanical properties change with temperature and degree of cure. Finally, differential finite element (FE) codes were devised and incorporated into ABAQUS with a UMAT subroutine, which was then utilized to model the evolution of residual stress in composite laminates exposed to different curing cycles and validated through experimental verification.

## 2. Multiscale Modeling Process

### 2.1. Resin Cure Kinetics

The kinetics of the resin curing reaction was characterized using DSC. Resins undergo a series of phase transitions during the curing process, each accompanied by the release of exothermic heat due to polymerization reactions. From the DSC data, an empirical model that describes the cure rate of a resin can be developed by applying various kinetic models. In order to calculate the resin degree of cure α over time, it is necessary to measure the total heat $\Delta H_{whole}$ generated during the reaction process, as well as the instantaneous heat ΔH released:

(1) $$\alpha =\frac{\Delta H}{\Delta H_{whole}}.$$

The autocatalytic model effectively captures the cross-linking dynamics of polymer chains by incorporating two reaction orders. The autocatalytic model of an epoxy resin system is as follows [6,23]:

(2) $$\frac{d\alpha }{dt}=Ae^{\frac{-E_{\alpha }}{RT}}\alpha^{m}{\left(1-\alpha \right)}^{n}.$$

By applying the natural logarithm to both sides of Equation (2), we can obtain:

(3) $$\ln\left(\frac{d\alpha }{dt}\right)=\ln A+m\ln\alpha +n\ln(1-\alpha )-\frac{E_{\alpha }}{RT},$$

in which *α* represents the degree of cure, *T* is the temperature, *A* stands for the pre-exponential factor, $E_{\alpha }$ denotes the activation energy, *R* is the gas constant, and *m*, *n* characterize the reaction order of the cure kinetics. They are obtained from multivariable linear regression of the DSC data.

An *N*th-order kinetic model suggests that a single reaction order prevails during the curing process, and the rate equation is expressed as follows [24]:

(4) $$\frac{d\alpha }{dt}=Ae^{\frac{-E_{\alpha }}{RT}}{\left(1-\alpha \right)}^{n}.$$

The natural logarithm is calculated for both sides of Equation (4):

(5) $$\ln\left(\frac{d\alpha }{dt}\right)=\ln A+n\ln(1-\alpha )-\frac{E_{\alpha }}{RT}.$$

### 2.2. Cure-Dependent Modulus

The elastic modulus of the resin experiences significant development in response to changes in the degree of cure during manufacture. The instantaneous isotropic resin modulus is described by Bogetti [23] as follows:

(6) $$\left\{\begin{array}{l} E_{m}=E_{m}^{0}\;\;\;\;\;\;\;\;\;\;\;\alpha <\alpha_{gel}\\ E_{m}=\left(1-\alpha_{\mathrm{mod}}\right)E_{m}^{0}+\alpha_{\mathrm{mod}}E_{m}^{\infty }\;\alpha \geq \alpha_{gel}\;, \end{array}\right.$$

in which $\alpha_{\mathrm{mod}}=\frac{\alpha -\alpha_{gel}}{1-\alpha_{gel}}$, $E_{m}^{0}$ represents the resin modulus at the onset of the curing process, $E_{m}^{\infty }$ denotes the modulus of the fully cured resin, and $\alpha_{gel}$ is the degree of cure at the gel point (theoretically 0.63 for epoxy resin [17]).

The shear modulus is given by:

(7) $$G_{m}=\frac{E_{m}}{2\left(1+v_{m}\right)},$$

where the Poisson ratio of the resin is specified as [25]:

(8) $$v_{m}=\frac{1}{2}\left(1-\frac{E_{m}\left(1-2v^{\infty }\right)}{E_{m}^{\infty }}\right).$$

Furthermore, the orthotropic elasticity of the laminate at specified degree of cure and temperature can be described through a micromechanical model [26]. The properties of T700 carbon fiber and the cured epoxy resin are listed in Table 1, which provides detailed material characteristics essential for accurate modeling and analysis.

### 2.3. Viscoelastic Constitutive Model

During curing, the relaxation of residual stresses formed in composites can be characterized by a detailed viscoelastic constitutive equation that incorporates a hereditary integral. For situations involving variations in both temperature and degree of cure, the stress in a linearly viscoelastic material can be calculated with the equation below [16,27]:

(9) $$\sigma (t)=\int_{0}^{t}E(\alpha ,T,t-\tau )\frac{d}{d\tau }\left[\varepsilon^{total}(\tau )-\varepsilon^{tc}(\tau )\right]d\tau ,$$

where $\varepsilon^{tc}$ represents the free thermochemical strain resulting from alterations in both temperature and degree of cure. Composite materials exhibit thermal flow behavior, and the corresponding equation is written as [28]:

(10) $$\sigma (t)=\int_{0}^{t}E\left(\xi (t)-\xi^{\prime }(\tau )\right)\frac{d}{d\tau }\left[\varepsilon^{total\;}(\tau )-\varepsilon^{tc}(\tau )\right],$$

in which ξ and $\xi^{\prime }$, termed reduced times, are a time representation combining temperature and degree of cure and are described as:

(11) $$\xi (t)=\int_{0}^{t}\frac{1}{a_{T}(\alpha ,T)}dt^{\prime };\;\;\;\xi^{\prime }(\tau )=\int_{0}^{\tau }\frac{1}{a_{T}(\alpha ,T)}dt^{\prime },$$

where $a_{T}$ is the shift factor and enables the time–temperature superposition. Thermal expansion $\varepsilon^{th}$ and chemical shrinkage $\varepsilon^{ch}$ are the major source of the primary cause of non-mechanical strains $\varepsilon^{tc}$ which is expressed as:

(12) $$\left\{\begin{array}{l} \varepsilon^{tc}=\varepsilon^{th}+\varepsilon^{ch}\\ \varepsilon^{th1}=\alpha_{T_{1}}(T-T_{a})\\ \varepsilon^{th2}=\alpha_{T_{2}}(T-T_{a}). \end{array}\right.$$

The thermal expansion coefficients along the fiber direction $\alpha_{T_{1}}$ and the transverse direction $\alpha_{T_{2}}$ are defined as:

(13) $$\left\{\begin{array}{l} \alpha_{T1}=\frac{\alpha_{1f}E_{f}V_{f}+\alpha_{m}E_{m}V_{m}}{E_{f}V_{f}+E_{m}V_{m}}\\ \alpha_{T2}=\left(\alpha_{2f}+v_{12f}\alpha_{1f}\right)V_{f}+\left(\alpha_{m}+v_{m}\alpha_{m}\right)V_{m}, \end{array}\right.$$

in which $E_{f}$ and $E_{m}$ are the elastic moduli, $V_{f}$ and $V_{m}$ are the volume fractions, and $\alpha_{f}$ and $\alpha_{m}$ are the thermal expansion coefficients.

During the curing process, the volume of the resin decreases because of the chemical cross-linking reaction. The chemical shrinkage strain of an isotropic homogeneous resin can be characterized as follows:

(14) $$\varepsilon^{r}=\sqrt[{3}]{1+\alpha \nu_{re}}-1,$$

where $\nu_{re}$ is the volume resin reduction and equals to −0.03 [29]. This indicates that the effective thermal expansion and cure shrinkage of composites are impacted by the properties of their constituents and their volumetric proportions.

## 3. Model Verification and Numerical Implementation

### 3.1. Experimental Section

In this section, a UD prepreg suitable for high-temperature (180 °C) curing was used. The epoxy resin was tailored for high-temperature applications. The composite material was reinforced with T700 carbon fibers. The conventional DSC Q200 from TA Instruments (New Castle, DE, USA) was used to assess the exothermic flow of the epoxy prepreg during the manufacturing process and to determine the curing reaction parameters. In a nitrogen atmosphere, the uncured prepreg was placed in a DSC sample tray for dynamic scanning; the sample weight was approximately 18 mg. Subsequently, DSC was set at the different heating rates of 5 °C/min, 10 °C/min, 15 °C/min, and 20 °C/min within the temperature range of room temperature, up to 300 °C. Firstly, standard materials like indium were used for temperature calibration, recording their melting points and adjusting the instrument settings accordingly. Next, we conducted a heat flow calibration using a material with a known heat capacity, such as sapphire, and adjusted the heat flow settings. A stable baseline was established by running an empty crucible experiment. Finally, the calibration was verified with additional standard materials to ensure accuracy and precision.

The storage and loss moduli were evaluated by DMA-Q800 from TA Instruments (New Castle, DE, USA). A sample measuring 50×5×2 mm was tested in the 3-point bending mode. Before inserting the standard spline into the DMA, we accurately measured the spline using a vernier caliper and determined the average value. The calibrated device functioned across multiple cure cycles and heating rates in the 3-point bending mode with a testing frequency of 1 Hz and a heating rate of 5 °C/min, consistently maintaining an oscillation amplitude of 20 µm.

Directional strains in laminates during the curing reaction are measured using Fiber Bragg Grating (FBG) sensors. The conversion relationship among strain, temperature, and wavelength $\lambda_{B}$ is as follows [30]:

(15) $$\frac{\Delta \lambda_{B}}{\lambda_{B}}=\left(\alpha_{f}+\varsigma \right)\Delta T+\left(1-p_{e}\right)\Delta \varepsilon$$

**Figure 2:**
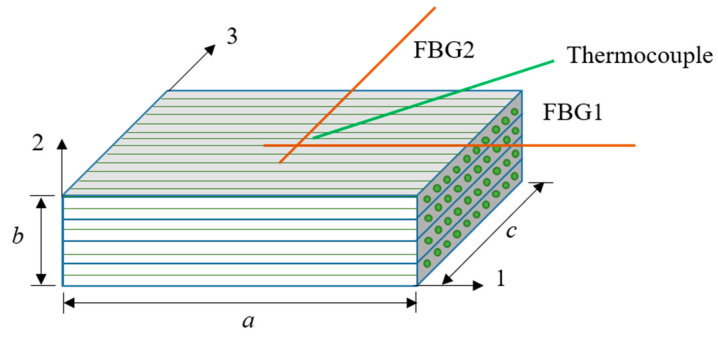
Location of FBG sensors and thermocouple for strain measurement.

In which $\alpha_{f}$ denotes the thermal expansion, and ς and $p_{e}$ are the thermo-optic and photo-elastic coefficients of the optical fibers. The coefficient $p_{e}$ equals 0.22 for silica optical fibers oriented in both directions. At the same time, a thermocouple was placed to compensate for the temperature response of the FBG sensors on the laminate. Figure 2 shows the placement of the FBG sensors and the thermocouple. They were placed in the center of a 4 mm thick UD laminate, indicating the material directions.

### 3.2. Numerical Model

A multi-scale model was established to simulate the stress–strain state during the laminate curing process. At the macroscopic level, stress, strain, temperature, and degree of cure of the material were determined. The unidirectional laminate was assumed to be a uniform transversely isotropic material, and we used a viscoelastic constitutive model to determine the macroscopic mechanical response throughout the curing process. At the microscale, the resin’s thermochemical properties were calculated. Temperature and degree of curing were calculated at the macroscale and transferred to the microscale. These calculations determined the corresponding thermochemical mechanical properties, which were fed back to the macroscopic level. 

**Figure 3:**
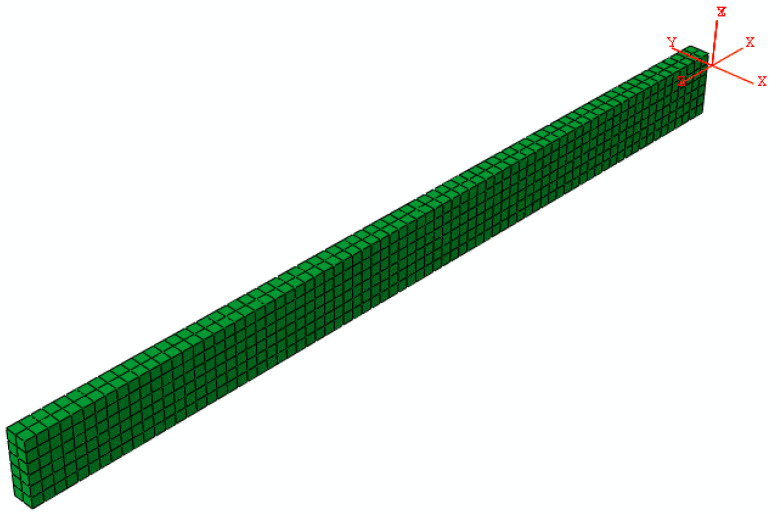
The geometry and mesh of the finite element model.

The mechanical model was executed and integrated into the commercial finite element software ABAQUS 2021, complete with the geometry and mesh configuration of the finite element model. The laminate plane was meshed using the C3D8T element. The user subroutines HETVAL and USFFLD were developed to investigate the curing kinetics of thermochemical models. Simultaneously, the user subroutines UMAT and UEXPAN were used to model the changes in stress and strain in relation to the degree of cure and temperature. A finite element model with 4 mm thick laminate boundary conditions was obtained following the guidelines provided in Figure 3.

**Figure 4:**
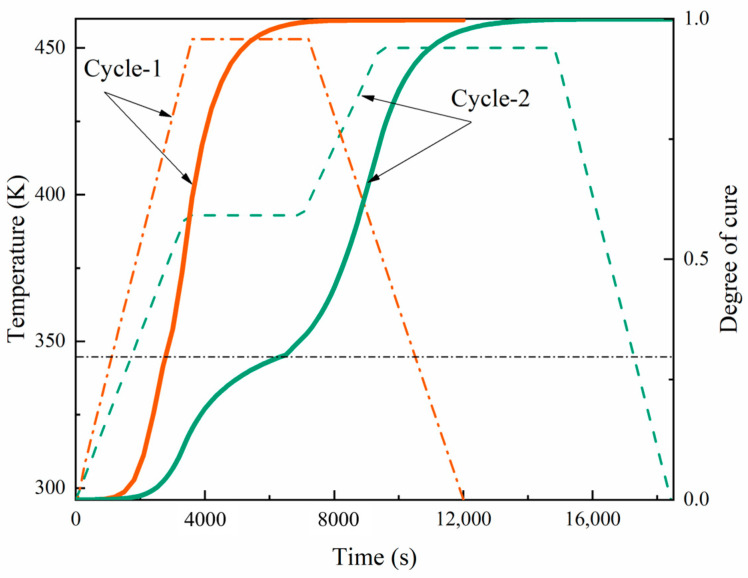
Development of the degree of cure in the central point of the laminate. During the curing process of thick laminates, the curing speed is different between the interior and the surface of the material. The temperature gradients cause inconsistent thermal expansion and contraction. This inconsistency can result in residual stress within the laminate, potentially causing warping, deformation, and even cracking of the laminate, thereby compromising its dimensional stability and structural integrity. Additionally, different degrees of cure between the inner and the outer layers can result in uneven crosslinking density, leading to disparities in the mechanical properties (such as strength and toughness) in different locations, thus reducing the overall performance and reliability of the material. Furthermore, uneven solidification during the curing process can introduce defects such as uncured areas or bubbles, increasing the risk of material failure during service. An optimized curing process is necessary to overcome these challenges, requiring real-time monitoring systems to ensure uniform curing and high-quality laminate performance.

The dashed line in Figure 4 shows the manufacturer’s recommended curing cycle. For thicknesses below 4 mm, curing cycle-1 is recommended, which involves heating from room temperature to 180 °C at a rate of 2.5 °C/min, holding this temperature for 1 h, and then cooling down to room temperature at a rate of 2 °C/min. For thicknesses greater than 4 mm, curing cycle-2 is recommended, initially heating up to 120 °C at a rate of 1.5 °C/min, maintaining this temperature for one hour, then increasing it to 180 °C at the same rate, holding it for 90 min, and finally returning to room temperature at a rate of 2.5 °C/min. The curves in the figure also show a comparison of the distribution of the degree of curing. With temperature changes, the curing rate in cycle-1 becomes more rapid, and the glass transition phase is less pronounced. However, for thicker laminates, this can lead to uneven curing. Using the *t*-test method, the t-statistic value was calculated to be 1.009, and the *p*-value was 0.320. Here, we compared the curing degrees of cycle-1 and cycle-2 at specific time points. At all time points, there was a small statistical difference in the overall solidification degree between the two cycles. The performance difference between the two cycles under real conditions could be evaluated through practical application testing.

## 4. Results and Discussion

### 4.1. Cure Kinetics

**Figure 5:**
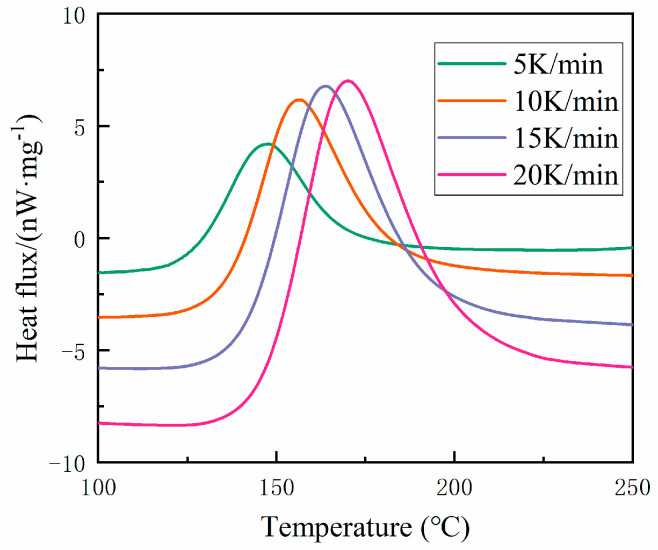
Heat flux based on the temperature at different heating rates.

**Figure 6:**
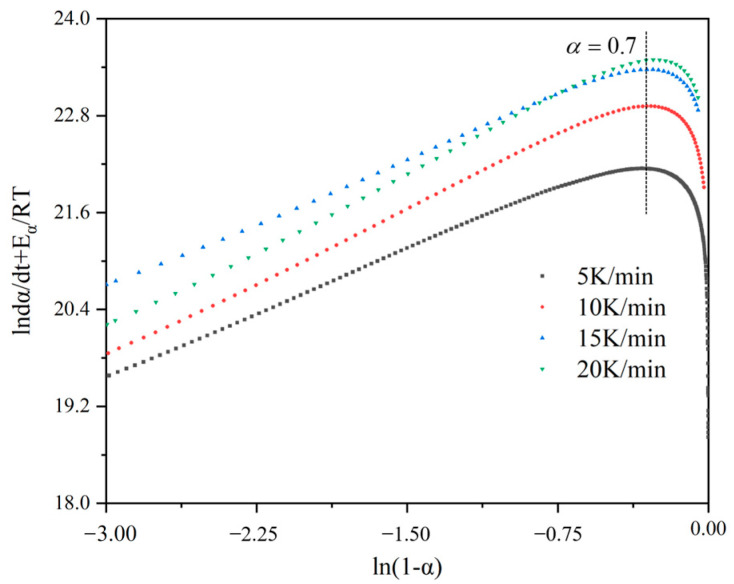
Fit relationship diagram for the Nth-order reaction kinetics model.

We examined the cure kinetics over a degree range from 0.05 to 0.9 to derive the related parameters. Both Nth-order and autocatalytic models were employed to analyze the cure kinetic parameters from the collected data. Initially, dynamic measurements were conducted using DSC at various heating rates (5 °C/min, 10 °C/min, 15 °C/min, 20 °C/min), and the heat flux curves consistently displayed an endothermic peak. Figure 5 shows this phenomenon at different heating rates. By employing various dynamic models, an empirical model depicting the curing rate can be derived from DSC data. The Nth-order kinetic model presumes a singular order reaction throughout the entire curing process. The cumulative data are graphed and linearly fitted to determine the reaction order. Figure 6 displays the curve obtained using an Nth-order dynamic model. It was observed that a linear relationship existed when the curing degree was below 0.7, but significant nonlinearity arose when the curing degree exceeded 0.7. This discrepancy suggests that when the curing degree surpasses 0.7, the Nth-order kinetic model fails to conform to Equation (4), thereby indicating that the Nth-order model cannot accurately represent the curing reaction process of the material. The Nth-order model began to fail at a degree of cure α of approximately 0.7, across all heating rates analyzed (5 K/min, 10 K/min, 15 K/min, and 20 K/min). At this threshold, the values of $\ln\left(\frac{d\alpha }{dt}\right)+\frac{E_{\alpha }}{RT}$ ranged from 22.2 to 23.0, depending on the heating rate. Beyond these points, significant deviations indicated the Nth-order model’s inadequacy for describing the curing process accurately.

**Figure 7:**
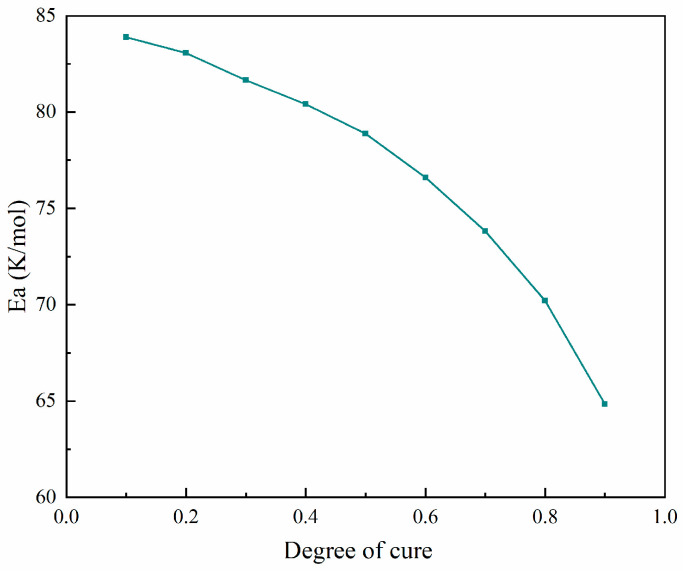
Relationship between activation energy and degree of cure for the curing process.

The activation energy $E_{\alpha }$ of the curing process showed a clear decreasing trend as the degree of cure increased from 0 to 1, as shown in Figure 7. Initially, at a degree of cure near 0, the activation energy was high, around 85 kJ/mol, indicating a significant energy requirement to initiate the curing reaction. As the degree of cure progressed to approximately 0.25, the activation energy decreased to about 80 kJ/mol and continued to drop gradually to around 70 kJ/mol at a degree of cure of 0.75. In the final phase, approaching full cure, the activation energy sharply declined to approximately 60 kJ/mol. This trend suggests that the curing process became progressively easier as the polymer network formed and crosslinking was facilitated, highlighting the importance of optimizing the curing conditions in different stages to ensure uniform and high-quality material properties.

**Figure 8:**
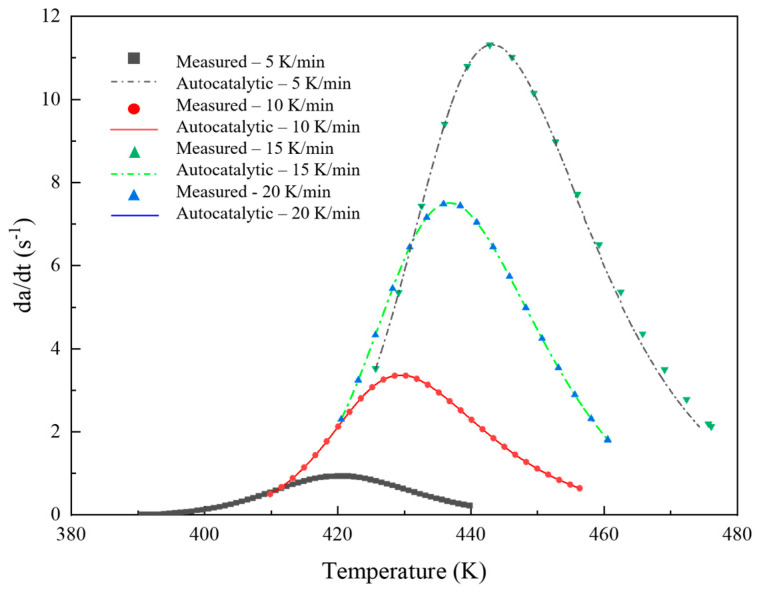
Comparison of the cure rate profiles at different heating rates.

**Figure 9:**
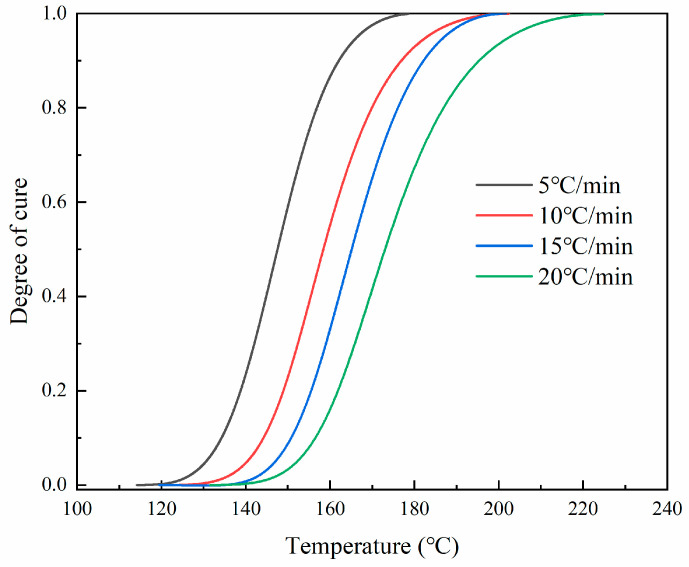
Degree of cure at different heating rates.

Nonlinear fitting was performed on other parameters in the model, and the curing kinetics parameters are shown in Table 2. Figure 8 shows the comparison between the experimental and the autocatalytic models. The high *R*^2^ values across all heating rates indicated that the autocatalytic model fitted the experimental data well, maintaining a high predictive accuracy. The slight decrease in *R*^2^ at higher heating rates suggests minor deviations, but overall, the model remained robust. It can be seen that the model and the experimental data were in good agreement in both early and late stages of the curing reaction. The autocatalytic model, which accounted for the concentration of resin and reaction products in calculating the reaction rate, could accurately predict the curing process of the prepreg system. Additionally, consistency was apparent, as shown in Figure 9: the temperature range where the curing degree curve increased coincided with the peak temperature on the reaction rate curve. As the temperature continued to rise, the degree of curing tended to saturate (close to 1), and the corresponding reaction rate curve started to decrease, reflecting the gradual completion of the reaction. The solidification progress was slow in the low-temperature zone, and the corresponding peak of the reaction rate curve appeared in a higher temperature range.

Using Table 3, we conducted a detailed statistical comparison. It involved examining the *R*^2^ values of both models at different heating rates and calculating additional fit quality indices by using RMSE, AIC, and BIC. Taking the heating rate of 5 K/min as an example, the comparison is clearly shown in Table 3. The autocatalytic model consistently provided a better fit at different heating rates. This was evidenced by higher *R*^2^ values and lower RMSE, AIC, and BIC values, as expected. The Nth-order model, with significantly lower *R*^2^ values, indicated a much poorer fit to the experimental data. This detailed statistical analysis highlighted the superiority of the autocatalytic model for describing the curing process.

### 4.2. Material Modulus Characteristics

**Figure 10:**
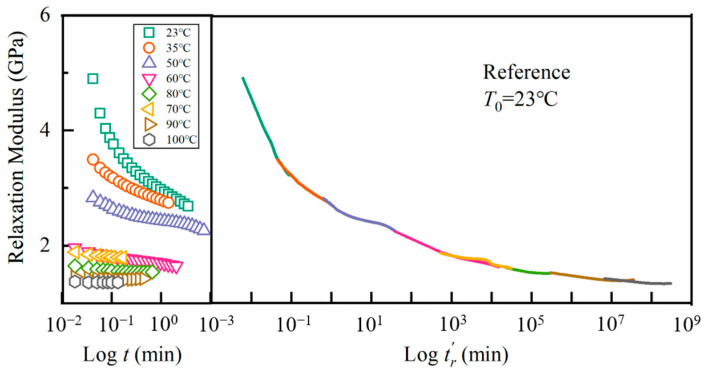
Relaxation modulus for the epoxy system and its master curve.

The mechanical properties of a resin change with polymerization and temperature. Understanding these changes is crucial for estimating residual stresses and the resins’ final properties. The dynamic storage modulus $E^{\prime }(w,T)$ is converted to the apparent tensile relaxation modulus $G(t_{r},T)$ as follows [31,32]:

(16) $$E^{\prime }(w,T)\approx G(t_{r},T)$$

in which $t_{r}$ is the relaxation time. On the left side of Figure 10, we show the relaxation modulus against time in a logarithmic scale at different temperatures. Except for the curve at the reference temperature *T*_ref_ = 23 °C, the other curves are horizontally shifted, tending to overlap. As depicted on the right side of this figure, a single smooth master curve against the reduced time was obtained. The smoothness of the curve confirmed the applicability of the time–temperature superposition principle to the apparent tensile relaxation modulus.

**Figure 11:**
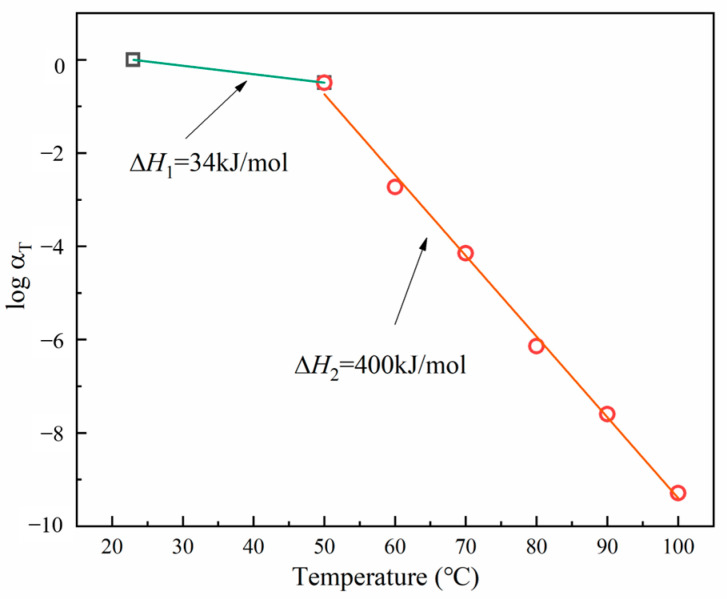
Shift factors for the relaxation modulus.

Here, we used the shift factor $\alpha_{T}$ of the Arrhenius equation at temperatures near or above the glass temperature:

(17) $$\log\alpha_{T}=\frac{\Delta H}{2.303R}(\frac{1}{T}-\frac{1}{T_{0}})$$

where *R* represents the gas constant, set at 8.314 × 10^−3^ [kJ/(K mol)]. The shift factors for the relaxation modulus *G* used in the master curve construction are shown in Figure 11. We set x=r indicating the relaxation. The shift factors for the tensile relaxation modulus aligned well with the Arrhenius equation with $\Delta H_{1}=34\;\mathrm{kJ}/\mathrm{mol}$ and $\Delta H_{2}=400\;\mathrm{kJ}/\mathrm{mol}$. Through their accurate fitting with the experimental data, reflecting the material physical behavior, it was proved that at low temperatures, a small activation energy corresponded to a middle thermal activation value, while a high activation energy at high temperatures indicated significant molecular mobility and rearrangement. These shift factors can construct an accurate master curve of the tensile relaxation modulus, which can then be used to analyze the relaxation behavior of materials over a wide temperature range.

**Figure 12:**
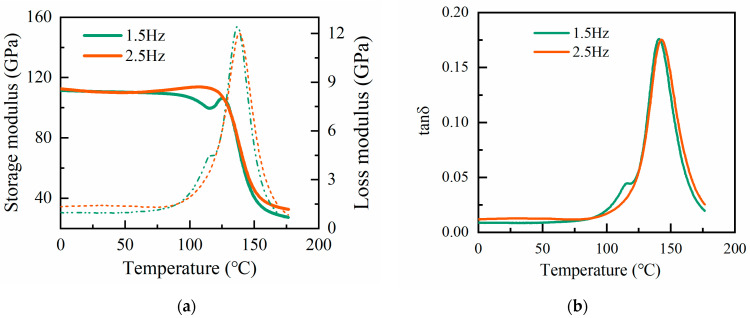
(**a**) Elastic modulus of the cured resin at different frequencies; (**b**) loss factor at different frequencies.

The fully cured samples were readied with a specific curing cycle for DMA analysis. Tension tests were conducted to evaluate how the elastic modulus of the specimens changed with temperature at varying frequencies. The temperature was incrementally raised, and isothermal pauses of 5 min were implemented to establish thermal equilibrium. Figure 12a shows the storage modulus and loss modulus of E of the cured resin. Figure 12b shows the loss factor tanδ in relation to temperature. The resin transformed from liquid to solid gel as the temperature increased. The storage modulus of the cured resin decreased at around 125 °C and tended to stabilize at around 170 °C. At temperatures above 180 °C, the resin was completely cured, and the modulus tended to stabilize. The loss factor remained low and constant at lower temperatures when the material was in its glassy state and rose at higher temperatures as the material became viscoelastic. The peak of the loss factor typically occurred near the glass transition temperature of the cured resin. Prior to vitrification, a significant variation was noted in the measured storage modulus as the frequency of the specimen increased. The experimental results indicated a difference in the storage modulus of 8 GPa, and the loss modulus was 0.6 GPa. After the vitrification of the resin, the difference between the storage modulus and the energy dissipation modulus at the two indicated frequencies was 5 GPa and 0.3 GPa.

### 4.3. Residual Stress and Strain Simulation

In this section, we describe the finite element verification of the proposed viscoelastic model and the numerical simulation of composite laminates that we conducted to study the residual stress distribution during the curing of a CFRP. The composite material studied was T700/epoxy resin unidirectional prepreg with a thickness of 4 mm. To facilitate the comparison with the experiments, the finite element model was set to four layers. Under the recommended curing cycle, the mechanical state of the center point (a/2, b, c/2) of the laminate in Figure 2 was studied.

**Figure 13:**
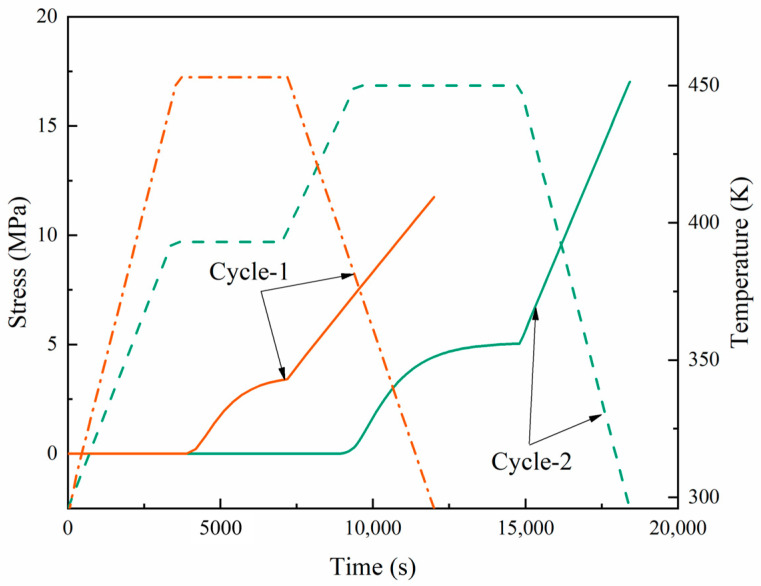
Development of longitudinal stress during the cure cycles in the examined laminates.

**Figure 14:**
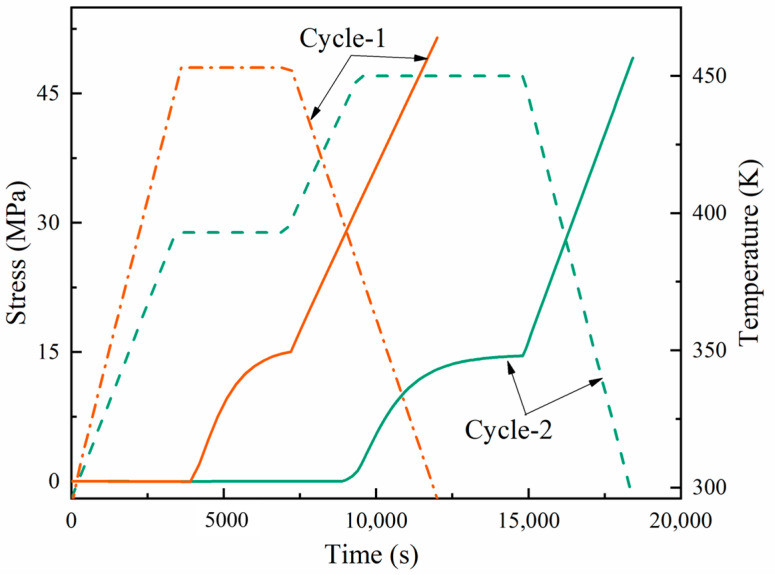
Development of transverse stress during the cure cycles in the examined laminates.

Figure 13 and Figure 14 present a dual-axis plot tracking the temperature and stress of a carbon fiber-reinforced composite over time through two curing cycles. Cycle-1 is represented in orange, and cycle-2 in green. The orange dashed line represents the curing temperature curve for cycle-1, while the solid line represents the residual stress. At a certain time, the corresponding stress depended on the temperature. The curing time for cycle-1 was 3.33 h, and that for cycle-2 was 5 h. The key aspects to note are the points of vitrification and transition to a gel state, which are significant in the curing process. In both cycles, the stress response to the thermal treatment was apparent; however, the stress in cycle-2 suggests a more complex interaction, possibly due to the response to the dual-stage heating process. Notably, the stress did not completely revert to its initial state as the temperature returned to baseline in both cycles, suggesting that residual stress remained in the material after curing.

In the initial stage of cycle-2 curing, lasting less than 60 min, due to the fast stress relaxation and chemical shrinkage at a low degree of cure, the stress slightly decreased with the increasing temperature in the first heating stage (compressive stress). In the first stage, the resin began to cure and contract, and the shrinkage rate and thermal expansion rate of the material were similar; so, the stress increased but not significantly. In the second stage of heating and holding, due to the chemical hardening effect, the stress increased. In the later stage of holding in the second stage, the stress changes tended to stabilize, which can be attributed to the fact that a balance between the complete solidification of the material and stiffness relaxation was reached. During the cooling stage, the slow relaxation and thermal expansion of the material in the second stage resulted in a significant increase in stress. In the curing processes of cycle-1 and cycle-2, the total residual stresses were 11.7 and 17 MPa, respectively. Due to the fact that the composite material underwent two heating and insulation stages in the two cycles, many cross-linking reactions occurred, resulting in high stress levels in the material. At the same time, the second heating stage allowed more unreacted resins to participate in the reaction, increasing the curing degree and material rigidity.

Compared to Figure 14, the unidirectional direction of composite fibers resulted in a higher modulus and greater strength in the longitudinal direction (fiber direction) during the curing process. At the same time, the fibers could also withstand higher loads. In the longitudinal cycle-1 and cycle-2, the total residual stresses were 51.4 MPa and 49.1 MPa, respectively, with little difference between the two cycles.

**Figure 15:**
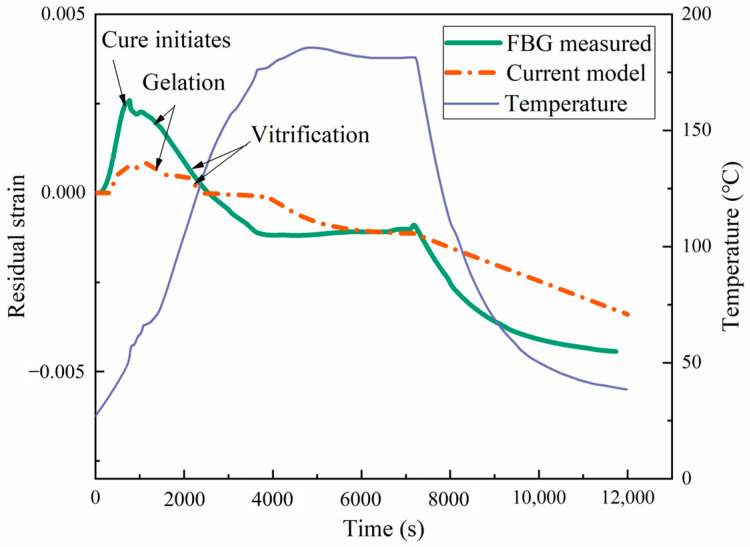
Longitudinal curing strain during the manufacturing process.

**Figure 16:**
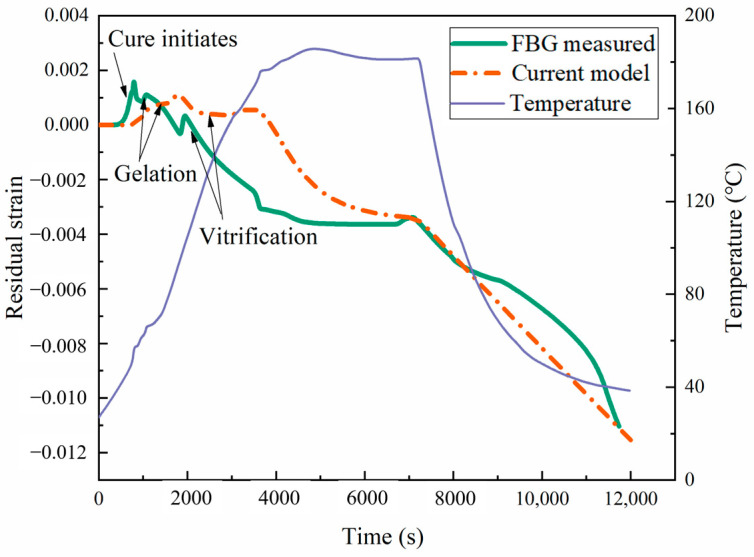
Transverse curing strain during the manufacturing process.

The comparison between the values measured for the FBG sensor and the numerical values of the curing shrinkage strain at the center of the laminate is shown in Figure 15 and Figure 16. In the actual experimental testing, an oven was used for curing, with pressure applied to ensure the curing behavior of the material with cycle-1. The numerical simulation results revealed a dynamic interplay between temperature and strain during the curing of the prepreg. Initially, thermal expansion caused the strain to increase to a peak as the temperature rose. Concurrently, the semi-cured resin began its transition into a liquid state. As the curing process began, the material polymerization reaction caused resin shrinkage, resulting in a sharp decrease in the material strain. In this stage, the shrinkage in the laminate attributable to the curing process superseded the thermal expansion strain. Due to the slow chemical reaction of the polymer chain, a small delay in strain variation was observed after vitrification. After the resin curing was completed, the strain increased twice, proportionally to the temperature increase caused by thermal expansion. Finally, as the resin cooled, volume contraction due to the reduced temperature led to a minimization of the strain. At the end of curing, the difference between the transverse strain (−4640 με) based on the proposed modulus and the experimentally measured transverse strain (−4438 με) was very small. Therefore, the proposed viscoelastic modulus model can accurately predict the curing strain.

## 5. Conclusions

We comprehensively characterized the residual strain observed during the curing process of CFRPs. Subsequently, the kinetics of this process was meticulously analyzed using both *N*th-order and autocatalytic models. The autocatalytic model demonstrated a closer alignment with practical applications, and the temperature range increase in the curing degree curve was consistent with the peak temperature in the reaction rate curve. The alignment between the autocatalytic model and its applications was based on its accurate prediction of the kinetic parameters, the consistency of the obtained results with the experimental data, and its ability to describe the mechanical properties of laminate. An empirical model combining the curing degree and temperature was used to describe the thermochemical properties of the cured prepregs. This model effectively captured the viscoelastic behavior and shift factors by using the principle of time–temperature superposition. Following resin vitrification, the differential observed between the storage modulus and the loss modulus at varying frequencies was quantified as amounting to 5 GPa and 0.3 GPa. Viscoelastic models are utilized to investigate the material manufacturing process, predicting and analyzing the residual stress and strain during the curing process. For laminates measuring 4 mm in thickness, the difference between the two cycles was minimal. Comparing the curing cycles under the same conditions, the measured curing strain and the simulated curing strain showed overall consistency. This viscoelastic model assumes uniform material properties and curing, which may not be accurate for thicker laminates. This can cause inaccuracies in predictions. Thus, experimental validation and better modeling techniques are necessary to accurately predict complex laminate behaviors.

## Figures and Tables

**Table 1:** Mechanical characteristics of T700 and completely cured epoxy.

Laminate	Material Properties	
T700	Elastic modulus	115 GPa
	$\alpha_{1f}$	−0.9 × 10^−6^/K
	$\alpha_{2f}$	7.2 × 10^−6^/K
	Poisson’s ratio	0.3
Epoxy	Thermal coefficient	5.27 × 10^−5^/K
	Volumetric shrinkage	0.29

**Table 2:** Cure kinetic parameters of autocatalytic model and Nth-order model.

Heating Rate/(K·min^−1^)	Autocatalytic Model				Nth-Order Model		
	ln(*A*)/min^−1^	*m*	*n*	*R* ^2^	ln(*A*)/min^−1^	*n*	*R* ^2^
5	23.26	0.55	1.28	0.997	21.53	0.179	0.49
10	23.96	0.5	1.41	0.991			
15	24.23	0.4	1.21	0.987			
20	24.39	0.39	1.43	0.988			

**Table 3:** Summary of the expanded comparison.

Model	*R* ^2^	RMSE	AIC	BIC
Autocatalytic	0.997	0.15	30.1	35.4
Nth-order	0.490	0.60	45.0	50.3

## Data Availability

The data are contained within the article.
